# Cross-examining candidate genes implicated in multiple system atrophy

**DOI:** 10.1186/s40478-019-0769-4

**Published:** 2019-07-24

**Authors:** Jared S. Katzeff, Katherine Phan, Sivaraman Purushothuman, Glenda M. Halliday, Woojin Scott Kim

**Affiliations:** Brain and Mind Centre & Central Clinical School, The University of Sydney, Sydney, NSW Australia

**Keywords:** Multiple system atrophy, α-Synuclein, COQ2, Susceptibility genes, GWAS, Parkinson’s disease

## Abstract

Multiple system atrophy (MSA) is a devastating neurodegenerative disease characterized by the clinical triad of parkinsonism, cerebellar ataxia and autonomic failure, impacting on striatonigral, olivopontocerebellar and autonomic systems. At early stage of the disease, the clinical symptoms of MSA can overlap with those of Parkinson’s disease (PD). The key pathological hallmark of MSA is the presence of glial cytoplasmic inclusions (GCI) in oligodendrocytes. GCI comprise insoluble proteinaceous filaments composed chiefly of α-synuclein aggregates, and therefore MSA is regarded as an α-synucleinopathy along with PD and dementia with Lewy bodies. The etiology of MSA is unknown, and the pathogenesis of MSA is still largely speculative. Much data suggests that MSA is a sporadic disease, although some emerging evidence suggests rare genetic variants increase susceptibility. Currently, there is no general consensus on the susceptibility genes as there have been differences due to geographical distribution or ethnicity. Furthermore, many of the reported studies have been conducted on patients that were only clinically diagnosed without pathological verification. The purpose of this review is to bring together available evidence to cross-examine the susceptibility genes and genetic pathomechanisms implicated in MSA. We explore the possible involvement of the *SNCA*, *COQ2*, *MAPT*, *GBA1*, *LRRK2* and *C9orf72* genes in MSA pathogenesis, highlight the under-explored areas of MSA genetics, and discuss future directions of research in MSA.

## Introduction

Multiple system atrophy (MSA) is a fatal neurodegenerative disease that is characterized by progressive autonomic failure, parkinsonism and cerebellar ataxia [23]. It is an adult onset disease with a mean onset age of 52–59 years old and an average survival time of 7–9 years from diagnosis [56, 71]. The annual incidence in the age group 50–99 years has been estimated at 3–4 cases per 100,000 person years [10, 70]. There are two main subtypes of MSA – a parkinsonian subtype (MSA-P) with striatonigral degeneration and a cerebellar subtype (MSA-C) with olivopontocerebellar degeneration [34, 74]. In Caucasian populations MSA-P cases generally outnumber MSA-C cases by 2:1–4:1, however, in Japan MSA-C predominate over MSA-P [40, 41, 83].

The major pathological hallmark of MSA is the presence of glial cytoplasmic inclusions (GCI) in oligodendrocytes [50, 57]. The major constituent of GCI is α-synuclein, and therefore MSA is classified as an α-synucleinopathy, a group of neurodegenerative diseases that also includes Parkinson’s disease (PD) and dementia with Lewy bodies (DLB) [5, 26, 81]. Apart from GCI, other cellular inclusions are present in MSA brain, including neuronal cytoplasmic inclusions, neuronal nuclear inclusions and less commonly glial nuclear inclusions [37, 57]. Although the molecular mechanisms of misfolding, fibrillation and aggregation of α-synuclein partly overlap with other α-synucleinopathies, the pathological pathway of MSA is unique in that the initial principal site for α-synuclein deposition is in the oligodendrocytes rather than neurons; α-synuclein is normally concentrated in nerve terminals. It appears that p25α, which is required for microtubule stability in oligodendrocytes, plays a role in the α-synuclein deposition [30, 52].

Currently, MSA is considered a sporadic disease as no causative genes have been identified, although a number of gene variants has been shown to be associated with increased risk of MSA. In one study, it was estimated that the heritability among common variants to be 2.09–6.65% [22]. In a few studies, certain susceptibility genes have been suggested to be familial, including *SNCA* and *COQ2* [2, 46]. Also, the ethnic variation in MSA subtype indicates there may be a genetic predisposition to the specific subtypes of MSA [53]. The purpose of this review is to bring together available data to canvass the genetics of MSA. We will discuss the genes that are potentially associated with MSA – *SNCA*, *COQ2*, *MAPT*, *GBA1*, *LRRK2* and *C9orf72* – and recent findings from the MSA GWAS study [65].

### *SNCA* – α-Synuclein

The relevance of α-synuclein in MSA pathogenesis is of considerable focus [86]. α-Synuclein is encoded by the *SNCA* gene located on 4q22.1 [14]. It is concentrated at presynaptic terminals of neurons, where it is postulated to facilitate synaptic activity [14, 17, 59]. In MSA, however, α-synuclein is present in aggregated form as a major component of GCI in oligodendrocytes. Aggregation of α-synuclein appears to cause decreases in the level of neurotrophic factors in oligodendrocytes, reducing the ability of oligodendrocytes to provide axonal stability [79]. It also appears to impact on the ability of oligodendrocytes to regenerate and repair [88].

Mutations in *SNCA* (A30P, E46K, H50Q, G51D and A53T) are known to cause PD [4, 38, 42, 61, 91]. However, no causal *SNCA* mutations for MSA have been found to date, and the association between variant α-synuclein and MSA is still unproven. Several studies have investigated the inheritance of *SNCA* variants in families with MSA [38, 39, 58]. A study of a British family with the *SNCA* G51D variant revealed neuropathological hallmarks of both MSA and PD [38]. Similar hallmarks were present in two other cases with the same variant [39]. These findings suggest that G51D could be relevant to MSA as well as PD, although it has not been found in cases with only MSA pathology. Similarly, a Finnish family with *SNCA* A53E variant showed neuropathological hallmarks of both MSA and PD [58]. It should be noted that deposition of α-synuclein in oligodendroglia occurs with greater disease severity and duration in PD [28], suggesting that these mutations may be more aggressive PD mutations rather than relevant for the initiation of MSA. At this stage we cannot rule out the possibility of coexistence of the two diseases. Hence, at this stage, it is difficult to conclude if there is a causal link between *SNCA* and MSA.

The association of *SNCA* single nucleotide polymorphisms (SNPs) to MSA has also been studied. Two SNPs, rs3857059 and rs11931074, were found to be significantly associated with increased risk of MSA in an European population [67]. A further two SNPs, rs3822086 and rs3775444, were found to be significantly associated with increased risk of MSA in a different European population [2]. Interestingly, the latter study also found that rs3822086 was significantly associated with MSA-C [2]. In contrast, rs3822086 and rs3775444 were not significantly associated with MSA in a Chinese population[15].

 Overall, while current evidence suggests that specific *SNCA* SNPs are associated with increased risk of MSA in certain populations, no evidence exists for a causal relationship between *SNCA* and MSA, albeit PD/MSA or MSA-like pathology.

### *COQ2 –* coenzyme Q2 polyprenyltransferase

*COQ2* encodes the enzyme coenzyme-Q2-polyprenyltransferase in the biosynthetic pathway of coenzyme Q_10_. Coenzyme Q_10_ is an integral part of the mitochondrial electron transport chain; it transfers electrons from complex I and II to complex III [21]. Deficiencies in coenzyme Q_10_ cause mitochondrial dysfunction, oxidative stress and reduced ATP synthesis [62]. Polymorphisms in *COQ2* have been associated with MSA in several studies. In one study, functionally-impaired *COQ2* V393A variant was shown to be associated with sporadic MSA in a Japanese population [46]. This association was supported by a meta-analysis in a subsequent study, in which V393A was shown to be associated with increased risk of MSA in Han Chinese, Japanese and possibly broader East Asian populations [93]. In contrast, another study showed that V393A was not associated with MSA in 133 Japanese MSA patients [77]. Likewise, V393A was not associated with MSA in Korean MSA patients [35]. Apart from V393A, other *COQ2* variants (e.g. L25 V, M128 V, R173H, L402F, A32A and N386I) have been identified in various populations [16, 46, 77, 85]. The pathological link between *COQ2* and MSA was strengthened when it was discovered that plasma coenzyme Q_10_ levels were lower in MSA patients compared to healthy controls [48]. Furthermore, coenzyme Q_10_ levels were found to be lower in the cerebellum, along with increases in mitochondrial dysfunction and oxidative stress, in MSA cases compared to controls [7]. Interestingly, these changes occurred in MSA cases in the absence of any *COQ2* variants associated with MSA [7]. Overall, current evidence cannot fully exclude that specific *COQ2* variants are associated with MSA in only certain populations or ethnic groups, but that this pathway may be vulnerable to MSA. Further research is required to determine how *COQ2* may contribute to MSA pathogenesis and to reconcile the differences in different ethnic groups.

### *MAPT* – microtubule associated protein tau

*MAPT* is located on chromosome 17q21.31 and encodes the protein tau [9]. The role of tau in microtubule assembly and stability has long been established [84]. When phosphorylated, tau forms aggregates and detaches from the microtubules causing microtubule instability and degradation [9, 36]. Tau has been implicated in numerous neurodegenerative diseases including Alzheimer’s disease (AD), frontotemporal dementia (FTD), corticobasal degeneration (CBD), progressive supranuclear palsy (PSP), Pick’s disease (PiD) and DLB, all of which are considered as either primary or secondary tauopathies [25, 82]. There is some evidence that tau could be involved in MSA pathogenesis. Although tau is present in GCI [12], it is unclear whether this is due to tau playing an active role in MSA pathogenesis or whether this occurs downstream in the disease process.

Two haplotypes have been identified for *MAPT*. The H1 haplotype is associated with increased risk of PSP, CBD and PD [6, 18, 90], whereas the H2 haplotype rs870723-G allele is associated with decreased risk of late-onset AD [3]. The *MAPT* haplotypes also appear to be associated with MSA risk. In a study consisting of 61 pathologically-confirmed cases of MSA, the H1 haplotype was shown to be a risk factor for MSA [80]. In another study consisting of 127 pathologically-confirmed cases of MSA, H2 and H1E were associated with decreased risk of MSA, whereas H1x and H1J were associated with increased risk of MSA [44]. There are six isoforms of tau that exist in the human brain, and different isoforms have been shown to have different pathological effects. Increased levels of the 4R isoform have been implicated in FTD [9, 11], whereas the 3R isoform has shown to be pathologically important in PiD, and both 3R and 4R deposit in AD [82]. Currently, the role of tau isoforms in MSA is unknown.

### *GBA1* – beta-glucocerebrosidase

*GBA1* encodes beta-glucocerebrosidase, which is an enzyme that cleaves glucocerebroside in lysosomes. Mutations in *GBA1* cause accumulation of glucocerebroside in cells and certain tissues, and can cause Gaucher’s disease (GD) [1]. *GBA1* mutations are also a common risk factor for PD and DLB [8, 92]. There is some evidence that *GBA1* could also be linked to MSA. Sequencing of coding regions and flanking splice sites of the *GBA1* gene in 969 MSA patients, sub-divided into Japanese, European and North American cohorts, revealed that the carrier frequency of GD-linked mutations in MSA was 1.75% across all groups [47]. Interestingly, there was a significant association between *GBA1* mutations and MSA-C [47]. In another study consisting of 17 pathologically-confirmed MSA and 82 AD cases, *GBA1* mutations – N370S, T369 M and R496 – were present in 4 MSA cases [73]. Importantly, the MSA group had a higher frequency of *GBA1* mutations compared to the AD group. However, in a study consisting of 108 pathologically-confirmed MSA cases, *GBA1* mutations were not associated with MSA [72]. And, the *GBA1* L444P mutation was not associated with MSA in 54 Chinese MSA patients [75]. Furthermore, no association was found with 54 lysosomal storage disorder genes in 375 MSA patients analyzed [60]. With these conflicting findings, there is still much uncertainty over whether *GBA1* is linked to MSA.

### *LRRK2 –* leucine-rich repeat kinase 2

*LRRK2* encodes an enzyme that functions as both a kinase and a GTPase in various cellular processes [33]. *LRRK2* variants are associated with increased risk of PD [55, 96]. *LRRK2* G2019S is the most common mutation and accounts for 3–10% of familial PD and 1–8% of sporadic PD in European populations [32]. *LRRK2* mutations, particularly G2019S, have been investigated in MSA, however, to date, no evidence of a causal link between *LRRK2* mutations and MSA has been found [54]. Furthermore, no associations were found between other *LRRK2* variants (R1628P and G2385R) and MSA in 318 MSA patients of Han Chinese origin [89]. However, a study consisting of 177 pathologically-confirmed MSA cases from American and British cohorts revealed that M2397 T was a protective haplotype for MSA [31]. In a very recent study, the G2019S *LRRK2* mutation was found in a neuropathology-confirmed MSA case [64]. Much more research is required to determine whether *LRRK2* is linked to MSA.

### *C9orf72 –* chromosome 9 open reading frame

Expansion of GGGGCC repeat sequence in the *C9orf72* gene has been implicated in both amyotrophic lateral sclerosis and FTD [20, 63]. *C9orf72* has been investigated in MSA, however no association, as yet, has been found between *C9orf72* and MSA. In a cohort of 100 British and American pathologically-confirmed MSA cases, there was no association between *C9orf72* expansion and MSA [68]. This finding was verified in subsequent studies of Chinese MSA cases [13, 76], European and American MSA cases [69]. Based on current evidence, it appears that *C9orf72* is not linked to MSA.

### Other MSA susceptibility genes

A number of other genes has been investigated in MSA, all of which have not been substantially verified. One of the genes examined is *CHCHD2*, which encodes coiled-coil-helix-coiled-coil-helix domain containing 2 protein that is involved in mitochondrial metabolism. *CHCHD2* V66 M mutation was identified in a MSA patient of Italian heritage [51]. The function of this mutation is unknown. In another study consisting of 89 Chinese MSA patients, it was shown that no *CHCHD2* variants were found and there were no associations between *CHCHD2* and MSA [87]. Two recent studies examined the CAG repeat expansion length of spinal cerebellar ataxia (*SCA*) genes in MSA. In one, it was reported that the *SCA1* CAG expansions were more common in MSA-C compared to MSA-P or controls in an Italian cohort [49]. In another, it was reported that there were no association between *SCA1* CAG expansion and MSA in Chinese MSA patients, rather ataxin-2 (*ATXN2*) was suggested as a risk factor for MSA [95]. The rs1799964 SNP in tumor necrosis factor-α (*TNF-α*) and the rs16944 SNP in interleukin1β (*IL1β*) have also been suggested to be potential risk factors for MSA in Han Chinese population [94].

### Genome wide association study in MSA

In 2016, a genome-wide associated study (GWAS) comprising 918 MSA cases and 3,864 controls of European ancestry was carried out [65], which is the largest genetic study into MSA to date. No genes were found to be significantly associated (*p* < 5 × 10^− 8^) with MSA after stringent multiple corrections. However, there were four genes with a *p* value < 1 × 10^− 6^; these were F-box protein other 47 (*FBXO47*), elongation of very long-chain fatty acid 7 (*ELOVL7*), endothelin-1 (*EDN1*) and *MAPT* [65]. *MAPT* was the only gene that has been previously implicated in MSA. Neither *SNCA* nor *COQ2* was found to be significantly associated with MSA. These results suggest that genes other than those previously associated with neurodegeneration could be involved in MSA. The SNPs identified from the GWAS were tested in another study consisting of 906 MSA cases and 941 controls of Chinese origin, however no significant association was found between these SNPs and MSA [27], once again indicating potential ethnic differences in MSA genetics. It is important to note that in both of these two studies, not all of the MSA cases analyzed were pathologically confirmed.

### Copy number variation in MSA

Copy number variations (CNVs) are DNA structural rearrangements, such as deletions, duplications, inversions and translocations, all of which can affect gene dosage [45]. The role of CNVs in the pathomechanisms of AD [19] and PD [43, 78] have been extensively studied. The first indication of a link between CNVs and MSA came from a Japanese study, in which it was reported that 32% of MSA patients examined were heterozygous for deletions in the Src homology 2 domain containing-transforming protein 2 *(SHC2)* gene, suggesting that *SHC2* copy number is important in MSA [66]. In contrast, however, it was found that changes in *SHC2* copy number were insignificant in a group of American MSA patients [24]. A recent study of genome-wide CNV in a Japanese cohort identified 311 CNVs related to MSA, of which three were significantly different in MSA compared to controls [29]. These CNVs were located on CTD small phosphatase-like *(CTDSPL),* polypeptide *N*-acetlgalactosaminyltransferase-like 6 *(GALNTL6)* and small nuclear ribonucleoprotein polypeptide N (*SNRPN).* Interestingly, all of these CNVs were located in introns, suggesting that the control of expression of these genes is at the level of transcription.

## Conclusion and future directions

In this review, we have cross-examined the genes and genetic mechanisms that are possibly linked/implicated in MSA. It is clear from this and other reviews that the genetic etiology of MSA is poorly understood, and there is no general consensus on genes that increase the susceptibility to MSA. This is due to a few recurring technical issues. Firstly, in most studies the sample size was relatively small with inadequate statistical power to see common gene variants with small effect sizes. Secondly, much of the genetic findings were dependent on ethnicity or geographical regions, suggesting a variety of more recent influences causing MSA associated gene variants. Thirdly, only some studies were carried out on pathologically-confirmed cases of MSA knowing that clinical diagnosis is still poor. Fourthly, associations with different progressions/treatments may have influenced results as only a single time point (i.e. end stage) in the disease has been investigated. Many of these issues have been borne from the fact that MSA is a rare disease and the difficulty in recruiting brain donors, especially in Asian countries. Much research is required to properly understand how the susceptibility genes are involved in MSA pathogenesis and progression, or whether they are involved at all. A larger GWAS comparing Caucasian and Asian cohorts that are pathologically confirmed is required to accurately determine genes that are ethnicity specific. Under-explored areas of MSA genetics include genome-wide CNV screening, gene isoform differences, and the role of different gene haplotypes. Future studies should also include, not only the genes that are normally linked to neurodegeneration, but genes in other pathological pathways, particularly those involved in oligodendroglial function and in mitochondrial dysfunction.
